# Tjap1/Pilt Is a cis-Golgi-Associated Protein Required for Golgi Integrity and Normal Drug Transporter Expression in Brain Microvascular Endothelial Cells In Vitro

**DOI:** 10.3390/pharmaceutics18060665

**Published:** 2026-05-28

**Authors:** Junqiao Mi, Annabelle Schoder, Aili Sun, Patrick Meybohm, Malgorzata Burek

**Affiliations:** 1 Department of Anesthesiology, Intensive Care, Emergency and Pain Medicine, University Hospital Würzburg, 97080 Würzburg, Germany; 2 Graduate School of Life Sciences, Julius-Maximilians-Universität Würzburg, 97074 Würzburg, Germany

**Keywords:** Tjap1/Pilt, brain microvascular endothelial cells, blood–brain barrier, cis-Golgi apparatus, Golgi integrity, drug transporters, solute carrier transporters

## Abstract

**Background:** Brain microvascular endothelial cells (BMECs) form the blood–brain barrier (BBB), a highly selective interface that restricts paracellular diffusion and regulates the transport of nutrients and drugs into the central nervous system via specialized transporters and receptors. Tight junction-associated protein 1 (Tjap1), also termed protein incorporated later into tight junctions (Pilt), has been localized to tight junctions (TJs) in epithelial cells and to the trans-Golgi network in fibroblasts; however, its expression, subcellular localization, and functional significance in BMECs are still unknown. **Methods:** We characterized Tjap1 subcellular localization in mouse and human BMEC cell lines as well as primary mouse BMECs by immunofluorescence with and without pharmacological Golgi disruption by treatment with Brefeldin A, Golgicide A or Pitstop 2. CRISPR/Cas9-mediated Tjap1 knockout cells were generated and examined with regard to their Golgi morphology using immunostaining. Tjap1 mRNA localization was examined by RNAscope in situ hybridization. Quantitative real-time PCR and Western blot was performed to assess the expression of BBB-associated efflux transporters, solute carrier transporters, and cellular receptors in control and Tjap1 knockout cells. **Results:** Tjap1 predominantly localized to the cis-Golgi compartment, co-localizing with Gm130 rather than Tgn38, and was absent from TJs in BMECs. Tjap1 knockout induced pronounced Golgi fragmentation BMECs. Importantly, Tjap1 knockout significantly downregulated mRNA-expression of Abcb1a, Abcb1b, Abcc4, Slc2a1, Slc7a1, Slc7a5 and Tfrc, while Abcg2 was upregulated. At the protein level, a decrease in the protein levels of Abcb1, Abcc4, Slc2a1, Slc7a1, and Tfrc was observed in Tjap1 knockout cEND cells. **Conclusions:** In BMECs, Tjap1 is a cis-Golgi-associated protein required for the structural integrity of the Golgi apparatus. Its deletion is associated with Golgi fragmentation and significant alterations in the mRNA and protein expression of drug transporters and receptors at the BBB. These findings identify Tjap1 as a candidate regulator of both Golgi architecture and the BBB transporter profile in vitro, with potential implications for modulating drug transport across the BBB.

## 1. Introduction

Brain microvascular endothelial cells (BMECs) form the primary cellular component of the blood–brain barrier (BBB), a highly selective interface that restricts paracellular diffusion and transcytosis, regulates vascular permeability, and helps maintain central nervous system (CNS) homeostasis [1]. Unlike peripheral endothelial cells, BMECs exhibit unique features that contribute to their exceptional barrier properties, including the presence of continuous tight junctions (TJs) dominated by transmembrane proteins such as claudin-5, occludin, and junctional adhesion molecules (JAMs), which interact with cytoplasmic scaffolding proteins including zonula occludens-1 (ZO-1), ZO-2, and ZO-3 to form a highly organized junctional complex [2]. In addition to restricting paracellular permeability, BMECs express a repertoire of ATP-binding cassette (ABC) efflux transporters, including P-glycoprotein (Abcb1a/b), breast cancer resistance protein (Bcrp/Abcg2), and multidrug resistance-associated proteins (Mrps/Abcc family), which actively pump substrates back into the blood and limit the brain penetration of a wide range of therapeutic agents [3,4]. BMECs also express solute carrier (SLC) transporters such as the glucose transporter Glut1 (Slc2a1), the L-type amino acid transporter Lat1 (Slc7a5), and the cationic amino acid transporter Cat1 (Slc7a1), which mediate the selective delivery of essential nutrients across the BBB [5]. Furthermore, cellular receptors including the transferrin receptor (Tfrc) and low-density lipoprotein receptor-related protein 1 (Lrp1) enable receptor-mediated transcytosis and have emerged as promising targets for delivering biologics and macromolecular therapeutics across the BBB [6,7]. Dysfunction of BMECs and disruption of TJ integrity have been implicated in numerous neurological disorders, including stroke, Alzheimer’s disease, multiple sclerosis, and brain tumors [8,9], and alterations in transporter expression can profoundly affect both CNS homeostasis and the efficacy of pharmacological interventions [10]. Understanding the molecular mechanisms that regulate the expression of these transporters in BMECs is therefore of fundamental importance for both BBB biology and CNS drug delivery.

The Golgi apparatus is a ribbon-like organelle comprising stacked flattened cisternae that are functionally divided into cis-Golgi network (CGN), cis-, medial-, and trans-cisternae, and trans-Golgi network (TGN) [11]. The structural integrity of the Golgi apparatus is maintained by matrix proteins, among which GM130, GRASP65, and GRASP55 are particularly important. GM130 is a cis-Golgi matrix protein that tethers ER-derived vesicles and maintains Golgi ribbon architecture, thereby supporting polarized trafficking, mitotic progression, and efficient glycosylation in BMECs [12]. GRASP65 and GRASP55 are Golgi reassembly stacking proteins localized at the cis-Golgi and medial/trans-Golgi compartments, respectively, that regulate Golgi ribbon linking, cisternal alignment, and localization of glycosylation enzymes [11]. The TGN serves as the primary sorting station at the exit face of the Golgi apparatus, where newly synthesized and processed proteins are segregated into distinct transport carriers destined for the plasma membrane, endosomes, or lysosomes [13]. In BMECs, the Golgi apparatus plays a critical role in the biosynthesis, post-translational modification, and trafficking of junction proteins and transporter trafficking that are essential for BBB integrity and function [14,15]. Golgi fragmentation, characterized by dispersal of the Golgi ribbon into ministacks or vesicular structures, impairs protein glycosylation and trafficking, leading to defective delivery of cell–cell junctional components and membrane transporters [16]. He et al. found that GM130 depletion in BMECs caused Golgi fragmentation accompanied by downregulation of occludin and claudin-5, resulting in BBB dysfunction [17]. Notably, Golgi fragmentation has been observed in neurodegenerative diseases and cerebrovascular disorders associated with BBB breakdown [14,18], suggesting that maintaining Golgi integrity is essential for brain endothelial cell function. However, the molecular regulators that maintain Golgi structure in BMECs remain incompletely understood.

One candidate regulator is Tight junction-associated protein 1 (Tjap1), originally termed Protein incorporated later into tight junctions (Pilt) and also known as Tight junction protein 4. Tjap1 was originally identified as a binding partner of human discs large (hDlg). Kawabe et al. first characterized Tjap1 as a peripheral membrane protein at TJs in MTD-1A mouse mammary epithelial cells. Notably, Tjap1 was incorporated into TJs at a late stage during the wound-healing process, following the initial formation of claudin-based junctional strands. In addition, Kawabe et al. observed Tjap1 staining at perinuclear regions corresponding to the Golgi complex, suggesting potential involvement in vesicle trafficking between the Golgi apparatus and TJs [19]. Subsequently, Tamaki et al. identified Tjap1 as an Arf6-binding protein and demonstrated its predominant localization at the trans-Golgi network in NIH3T3 fibroblasts, where its knockdown causes Golgi fragmentation, positioning it as a Golgi-associated regulator of Golgi structure [20].

Despite these advances, the expression, subcellular localization, and functional significance of Tjap1 in BMECs remain completely unexplored. Importantly, discrepancies exist between the reported localizations of Tjap1 in different cell types: Kawabe et al. demonstrated TJ localization in epithelial cells, whereas Tamaki et al. showed predominant trans-Golgi localization in NIH3T3 fibroblasts, noting that their antibody failed to label TJs in various epithelial cell types. Given that BMECs possess unique junctional composition and regulatory mechanisms distinct from peripheral epithelial cells, it remains unclear whether Tjap1 exhibits similar localization patterns and roles in BMECs. In the present study, we utilized three models of the brain endothelium (cEND, hCMEC/D3, and primary mouse BMECs), together with the human intestinal epithelial cell line Caco-2 as a reference model for non-CNS barrier, to determine the subcellular localization of Tjap1. We also examined whether Tjap1 contributes to the structural and functional integrity of the Golgi apparatus in BMECs, and whether the loss of Tjap1 affects the expression of drug transporters and cellular receptors. Our results demonstrate that, in BMECs, Tjap1 is predominantly localized within the cis-Golgi compartment rather than in the TJs; moreover, the absence of Tjap1 is accompanied by pronounced fragmentation of the Golgi apparatus, and changes in the mRNA and protein expression of key drug transporters and receptors.

## 2. Materials and Methods

### 2.1. Isolation and Culture of Primary Brain Endothelial Cells

Primary brain microvascular endothelial cells were isolated from murine brains exactly as described previously [21]. Briefly, the isolated mouse brains were immersed in working medium on ice followed by removal of cerebellum, olfactory bulb and meninges. The homogenate was adjusted to 10 mL with working medium, centrifuged at 1350× *g* for 5 min at 4 °C, and the pellet was resuspended in 18% dextran solution and vortexed to obtain a homogenous suspension. After centrifugation at 6080× *g* for 10 min at 4 °C, the myelin-rich supernatant and fluffy top layer were carefully removed, and the microvessel-enriched pellet was resuspended in pre-warmed digestion medium containing collagenase/dispase, DNase I, and Tosyl-L-lysyl-chlormethan-hydrochlorid (TLCK) and digested for 1 h 15 min at 37 °C with intermittent shaking. The cell suspension was then centrifuged at 1350× *g* for 5 min at room temperature (RT), washed once in warm Dulbecco’s Phosphate-Buffered Saline (DPBS). The final pellet was resuspended in full endothelial growth medium, and the cell suspension was seeded onto collagen IV–coated wells of 6-well plate, optionally supplemented with puromycin (8 µg/mL for the first 2 days) to select against contaminating non-endothelial cells. Primary mouse brain endothelial cells reached confluence within approximately one week and were used up to six passages. The identity and purity of the primary brain endothelial cells were validated by immunostaining for CD31, claudin-5, occludin, ZO-1 and VE-cadherin. The experiments were designed, performed, and reported according to the guidelines [22]. We used 6–8 weeks old male C57Bl/6N mice, purchased from Charles River Laboratories (Sulzfeld, Germany).

### 2.2. Cell Culture

cEND cells were isolated and immortalized as previously described [23,24]. The cells were grown in Dulbecco’s Modified Eagle’s Medium (DMEM; #D5796, Sigma-Aldrich, St. Louis, MO, USA) supplemented with 10% fetal bovine serum (FBS) and 1% penicillin/streptomycin in 37 °C and 5% CO_2_ incubator on plates coated with 0.5% gelatin. Human cerebral microvascular endothelial cell-line (hCMEC/D3, #SCC066, Merck Millipore, Darmstadt, Germany) cells were cultured with ECM cell medium (#1001, ScienCell Research Laboratories, Carlsbad, CA, USA) at 37 °C and 5% CO_2_ incubator on plates coated with collagen I. The Caco-2 cell lines were acquired from ATCC (#HTB-37, Manassas, VA, USA). The cells were cultured in Minimum Essential Medium (MEM, #51200038, Thermo Fisher Scientific, Waltham, MA, USA) supplemented with 10% FBS, 2 mM L-Glutamin, 1% Non-Essential Amino Acids (NEAA) and 1% penicillin/streptomycin in 37 °C and 5% CO_2_ incubator on plates coated with 0.5% gelatin. The medium was replaced three times a week.

### 2.3. Generation of Tjap1 Knockout and Knockdown Cells

For Tjap1 knockout (KO) cell line transfection, the cEND cells were seeded on 6-well plates at a density of 1 × 10^5^ cells/well and cultured overnight. At 70–80% confluence, cells were transfected with Pilt Double Nickase Plasmid (sc-428717-NIC, Santa Cruz Biotechnology, Dallas, TX, USA) or Control Double Nickase Plasmid (sc-437281, Santa Cruz Biotechnology, Dallas, TX, USA) using Lipofectamine™ 3000 Transfection Reagent (#L3000008, Thermo Fisher Scientific, Waltham, MA, USA) according to the manufacturer’s instructions for 48 h. The hCMEC/D3 cells were seeded on 6-well plates at a density of 1 × 10^5^ cells/well and cultured overnight. At 70–80% confluence, cells were transfected with Pilt Double Nickase Plasmid (sc-413980-NIC, Santa Cruz Biotechnology, Dallas, TX, USA) or Control Double Nickase Plasmid (sc-437281, Santa Cruz Biotechnology, Dallas, TX, USA) using Lipofectamine™ 3000 Transfection Reagent according to the manufacturer’s protocol for 48 h. Positive clones were selected with 4 µg/mL puromycin for 4–6 weeks. The knockout efficiency was determined by immunofluorescence staining or Western blotting.

For Tjap1 knockdown (KD) cell line transfection, the cEND cells were seeded on 6-well plates at a density of 1 × 10^5^ cells/well and cultured overnight. At 70–80% confluence, cells were transfected with Pilt shRNA Plasmid (sc-152265-SH, Santa Cruz Biotechnology, Dallas, TX, USA) or Control shRNA Plasmid (sc-108060, Santa Cruz Biotechnology, Dallas, TX, USA) using Lipofectamine™ 3000 Transfection Reagent according to the manufacturer’s instructions for 48 h. Stably transfected clones were selected with 4 µg/mL puromycin for 4–6 weeks. Knockdown efficiency was confirmed by PCR.

### 2.4. Immunofluorescence Staining

After fixation in 2% formaldehyde for 15 min at RT, the cEND cell line, hCMEC/D3 cell line, primary mouse brain endothelial cells, and Caco-2 cell line were permeabilized in 0.1% Triton X-100 in DPBS for 5 min at RT. After permeabilization and blocking, the cells were then exposed to primary antibodies [i.e., Rabbit anti-Tjap1 (1:500, #PAB22406, Abnova, Taipei, Taiwan), Mouse anti-Tjap1 (1:250, #68495-1-Ig, Proteintech, Rosemont, IL, USA), Mouse anti-claudin-5 (1:500, #35-2588, Thermo Fisher Scientific, Waltham, MA, USA), Mouse anti-ZO-1 (1:500, #33-9100, Thermo Fisher Scientific, Waltham, MA, USA), Mouse Anti-E-Cadherin (1:250, #610182, BD Biosciences, San Jose, CA, USA), Mouse Anti-Gm130 (1:250, #610822, BD Biosciences, San Jose, CA, USA), Mouse Anti-Tgn38 (1:250, #610898, BD Biosciences, San Jose, CA, USA; for human cell models hCMEC/D3 and Caco-2), Mouse Anti-Tgn38 (1:100, #sc-166594, Santa Cruz Biotechnology, Dallas, TX, USA; for mouse cell models cEND and primary mouse BMECs), Mouse Anti-Grasp65 (1:250, #sc-374423, Santa Cruz Biotechnology, Dallas, TX, USA), Rabbit Anti-Grasp65 (1:250, #10747-2-AP, Proteintech, Rosemont, IL, USA) and Rabbit Anti-Grasp55 (1:500, #10598-1-AP, Proteintech, Rosemont, IL, USA)] overnight at 4 °C. Cells were then incubated with Alexa Fluor-conjugated secondary antibodies for 1 h at RT in the dark. To stain the nuclei, the cells were mounted with ProLong^®^ Gold Antifade Reagent with DAPI. Images were screened using a fluorescence microscope (Microscope Axio Imager.M2, Carl Zeiss, Jena, Germany). For each immunofluorescence experiment, negative controls were performed in parallel to confirm the absence of non-specific staining. Furthermore, the specificity of the Tjap1 antibodies was confirmed by the complete loss of Tjap1 signal in Tjap1 knockout cell lines. Pearson’s correlation coefficient was calculated using the Coloc 2 plugin in ImageJ (version 1.54p, National Institutes of Health, Bethesda, MD, USA), with thresholds set automatically by Costes regression and the Costes significance test applied to each image. Identical parameters were used across all conditions.

### 2.5. Western Blot Analysis

Western blot was performed as previously described [8,24,25]. Briefly, total protein concentration was quantified by BCA Protein Assay Kit (#A55864, Thermo Fisher Scientific, Waltham, MA, USA) prior to loading, and 20 μg of protein per lane was loaded. Protein samples were separated in NuPage 4–12% Bis-Tris-Gel (#NP0336BOX, Thermo Fisher Scientific, Waltham, MA, USA). The proteins were transferred to PVDF membranes, and the membranes were then incubated with primary antibodies. Primary antibodies were diluted in DPBS containing 5% (*w*/*v*) non-fat milk. The following primary antibodies were used: Rabbit anti-Tjap1 (1:1000, Abnova, #PAB22406, Taipei, Taiwan), Mouse anti-P-glycoprotein (1:1000, #ALX-801-002-C100, Enzo Life Sciences, Farmingdale, NY, USA), Rat anti-Abcc4 (1:500, #ALX-801-039-C100, Enzo Life Sciences, Farmingdale, NY, USA), Mouse anti-Abcc5 (1:500, #sc-376965, Santa Cruz Biotechnology, Dallas, TX, USA), Rabbit anti-Abcg2 (1:1000, #ab207732, Abcam, Cambridge, UK), Rabbit anti-Slc2a1 (1:2000, #07-1401, Millipore, Darmstadt, Germany), Mouse anti-Slc7a1 (1:500, #sc-515782, Santa Cruz Biotechnology, Dallas, TX, USA), Rabbit anti-Lrp1 (1:2000, #ab92544, Abcam, Cambridge, UK), Mouse anti-Rage (1:500, #sc-365154, Santa Cruz Biotechnology, Dallas, TX, USA), Rabbit anti-Tfrc (1:1000, #ab65831, Abcam, Cambridge, UK), Rabbit anti-Tubulin (1:10,000, #ab197740, Abcam, Cambridge, UK), anti-β-Actin-Peroxidase (1:25,000, #A3854, Sigma-Aldrich, St. Louis, MO, USA), Mouse Anti-Hsp90 (1:2000, #60318-1-Ig, Proteintech, Rosemont, IL, USA) and Rabbit anti-Cox IV (1:1000, #4844, Cell Signaling Technology, Danvers, MA, USA). After incubation with respective secondary antibodies: anti-Rabbit IgG (1:3000, #7074s, Cell signaling Technology, HRP-linked Antibody, Danvers, MA, USA), anti-Mouse IgG (1:3000, #7076s, Cell Signaling Technology, HRP-linked Antibody, Danvers, MA, USA), and anti-Rat IgG (1:3000, #7077s, Cell Signaling Technology, Danvers, MA, USA), images were taken using an Enhanced Chemiluminescence solution and FluorChem FC2 Multi-Imager II (Alpha Innotech, San Leandro, CA, USA). For the subcellular fractionation experiment, Hsp90 and Cox IV served as cytoplasmic and membrane compartment markers, respectively, to confirm successful fractionation. For whole-cell lysate Western blots, β-actin or α-tubulin was used as the loading control. The intensity of protein bands was measured with ImageJ (version 1.54p, National Institutes of Health, Bethesda, MD, USA).

### 2.6. Membrane Protein Extraction

The membrane and cytoplasmic proteins were isolated and extracted using the Mem-PER™ Plus Membrane Protein Extraction Kit (#89842, Thermo Fisher Scientific, Waltham, MA, USA), according to the manufacturer’s instructions. In brief, the BMECs were washed twice with Cell Wash Solution by centrifugation at 300× *g* for 5 min. Permeabilization Buffer was then added to the cell pellet and incubated for 10 min at 4 °C with constant mixing. After centrifugation at 16,000× *g* for 15 min at 4 °C, the supernatant containing the cytosolic proteins was collected and stored at −80 °C. Subsequently, Solubilization Buffer was added to the remaining cell pellet and incubated for 30 min at 4 °C with constant mixing. After a second centrifugation at 16,000× *g* for 15 min at 4 °C, the supernatant containing the membrane proteins was collected and stored at −80 °C.

### 2.7. Drug Treatment

For pharmacological inhibition experiments, Golgicide A (#G0923, Sigma-Aldrich, St. Louis, MO, USA) and Pitstop 2 (#23885, Cayman Chemical, Ann Arbor, MI, USA) were each dissolved in DMSO to prepare 1 mg/mL stock solutions and stored at −20 °C. Brefeldin A (#B7450, Thermo Fisher Scientific, Waltham, MA, USA) was dissolved in DMSO at 5 mg/mL and stored under the same conditions. For treatment, 5 × 10^4^ cells from each cell line were seeded into 8-well Nunc™ Lab-Tek™ Chamber Slides (#177445, Thermo Fisher Scientific, Waltham, MA, USA). Upon reaching confluence, cells were treated with Brefeldin A at a final concentration of 5 μg/mL for 1 h in the incubator. For Pitstop 2 and Golgicide A, stock solutions were diluted in serum-free medium to final concentrations of 15 μM. Cells were incubated with Pitstop 2 for 15 min and with Golgicide A for 1 h. DMSO was used as a vehicle control at a final concentration of 0.1% (*v*/*v*), matched across all treatment conditions, and produced no detectable effect on Tjap1, GM130, or TGN38 staining compared to untreated cells. After treatment, cells were prepared for immunofluorescence staining as described above. Imaging analyses revealed that cell morphology remained unchanged after each treatment.

### 2.8. Wound Healing Assay

The wound healing assay was performed in accordance with the experimental approach of Kawabe et al. [19]. The objective was to determine whether the recruitment of Tjap1 to junctional regions of BMECs occurs during the restoration of cell junctions following a manual scratch. In brief, confluent primary mouse BMECs were digested with TrypLE™ Select Enzyme (#12563011, Thermo Fisher Scientific, Waltham, MA, USA), and 5 × 10^4^ cells were seeded into 8-wells-Nunc™ Lab-Tek™ chambers (#177445, Thermo Fisher Scientific, Waltham, MA, USA). After reaching confluence, the cells were manually scratched with the needle of 1 mL syringe (#9151141S, Omnican, B. Braun, Melsungen, Germany) and then cultured for 3, 6, and 12 h, followed by immunofluorescence staining.

### 2.9. RNAscope Analysis and Immunohistochemistry

RNAscope™ Multiplex Fluorescent Reagent Kit v2 (#323100, Advanced Cell Diagnostics (ACD), Newark, CA, USA) was used to perform RNA in situ hybridization, in accordance with the manufacturer’s instructions. In brief, 5 × 10^4^ cEND and primary mouse BMECs were seeded into 8-wells-Nunc™ Lab-Tek™ chambers (#177445, Thermo Fisher Scientific, Waltham, MA, USA). After confluence was reached, the slides were fixed on ice with cold 4% PFA solution for 60 min. The slides were then washed with DPBS, placed in the RNAscope EZ-Batch Slide Holder (ACD, Newark, CA, USA), and treated with RNAscope Hydrogen Peroxide. Then target retrieval using the steamer and treatment with PretreatPro Reagent for 30 min at 40 °C was performed. Probe hybridization was performed by incubating slides with RNAscope™ Probe—Mm-Tjap1 (# 1741341-C1, ACD, Newark, CA, USA) for 2 h in the HybEZTM II Oven (ACD, Newark, CA, USA). Hybridization of positive and negative controls (RNAscope 3-plex Positive-control Probe-Mm and RNAscope 3-plex Negative control Probe-Mm) was performed in parallel. For signal amplification, cells were incubated with AMP1 for 30 min, AMP2 for 30 min, and AMP3 for 15 min. HRP-based fluorescent detection was performed using a fluorescently tagged Opal dye 690 (1:1500, # FP1497001KT, Akoya Biosciences, Marlborough, MA, USA) for the detection of RNAscope probes. For immunocytochemistry, slides were blocked for 60 min at RT with 10% donkey serum and 0.1% Triton X in DPBS and then incubated overnight at 4 °C with a mouse anti-claudin-5 antibody (1:500, #35-2588, Thermo Fisher Scientific, Waltham, MA, USA). The following day, the slides were washed in DPBS and incubated for 1 h at RT with Goat anti-Mouse IgG (H + L) secondary antibody plus 488 (1:1000, #A55058, Thermo Fisher Scientific, Waltham, MA, USA). DAPI was applied to the slides before mounting with ProLong^®^ Gold Antifade Reagent. Images were taken using a fluorescence microscope (Microscope Axio Imager.M2, Carl Zeiss, Jena, Germany).

### 2.10. Quantitative Analysis of Golgi Morphology

Golgi morphology was quantified based on immunofluorescence images of GM130 in hCMEC/D3 and cEND cells, acquired using identical microscope settings. The analysis was performed in ImageJ (version 1.54p, National Institutes of Health, Bethesda, MD, USA) by individuals blinded to the experimental conditions: cells were segmented into ROIs using DAPI staining, and the number of discrete GM130-positive objects per cell was counted, following intensity thresholding using the “Analyze Particles” function. Identical parameters were applied to all images. To define a cell-line-specific fragmentation threshold, cells treated with DMSO (vehicle) or Brefeldin A were analyzed in parallel. DMSO-treated cells exhibited 6.75 ± 2.15 GM130-positive objects per cell in hCMEC/D3 and 7.63 ± 2.43 in cEND cells, whereas Brefeldin A-treated cells showed 20.57 ± 6.23 and 27.03 ± 7.51 objects per cell, respectively (mean ± SD; *n* = 30 cells per condition, pooled from three independent experiments). The fragmentation threshold was set at the upper 95% limit of the DMSO distribution (mean ± SD). On this basis, the following thresholds were established: ≥12 objects per cell for hCMEC/D3 and ≥13 objects per cell for cEND. Cells with an object count reaching or exceeding this threshold were classified as fragmented; cells with a lower object count were classified as compact. These thresholds served as the basis for all subsequent comparisons between the Tjap1 knockout condition and the control condition.

### 2.11. Glycoprotein Staining Assay

Glycoprotein staining was performed using the Pierce™ Glycoprotein Staining Kit (#24562, Thermo Fisher Scientific, Waltham, MA, USA) according to the manufacturer’s instructions. The total protein concentration was quantified prior to loading using a BCA Protein Assay Kit, and equal amounts of each sample were separated on a 10% (*w*/*v*) polyacrylamide gel. After electrophoresis, the gels were fixed in 50% methanol for 30 min and then washed twice with 3% acetic acid. Next, the gels were oxidized in Oxidizing Solution for 15 min and then washed three times with 3% acetic acid. Staining was performed for 15 min with Glycoprotein Stain. The gels were then treated with Reducing Solution for 5 min and thoroughly washed with 3% acetic acid and ultrapure water. Glycoproteins appeared as magenta bands. Images were acquired using FluorChem FC2 Multi-Imager II (Alpha Innotech).

### 2.12. Real-Time PCR

RNA isolation was performed as previously described [8,25,26,27]. Briefly, RNA was isolated using the RNA isolation kit NucleoSpin^®^ RNA (#740955.250, Machery-Nagel, Düren, Germany) according to manufacturer’s instructions. Total RNA (1 μg) was reverse transcribed using the High Capacity cDNA Reverse Transcription Kit (#4374966, Thermo Fisher Scientific, Waltham, MA, USA). The commercially available TaqMan probes Mm00503867_m1 (Tjap1) Mm00440761_m1 (Abcb1a), Mm00440736_m1 (Abcb1b), Mm00456156_m1 (Abcc1), Mm01226380_m1 (Abcc4), Mm01343626_m1 (Abcc5), Mm00496364_m1 (Abcg2), Mm01192270_m1 (Slc2a1), Mm01219060_m1 (Slc7a1), Mm00441516_m1 (Slc7a5), Mm01237129_m1 (Slc9a2) and Mm00451203_m1 (Slc5a1), Mm00464608_m1 (Lrp1), Mm01134790_g1 (Rage), and Mm00441941_m1 (Tfrc) were used with the TaqMan^®^ Fast Advanced Master Mix in the Thermo QuantStudio 7 Flex Real-Time PCR System. Calnexin (Canx) (Mm00500330_m1) was used as endogenous control. Relative gene expression was calculated using the comparative ΔΔCt method, as implemented in the QuantStudio™ Real-Time PCR Software v1.7.1 (Thermo Fisher Scientific, Waltham, MA, USA), which yields “relative quantification” (RQ) values for each target gene.

### 2.13. Statistical Analysis

GraphPad Prism 9.0 (GraphPad Software Inc., San Diego, CA, USA) was used for statistical analysis. All experiments were performed in three independent biological replicates (*n* = 3). The data are expressed as the mean ± standard deviation (SD). Pairwise comparisons between knockout and control conditions were performed using unpaired *t*-tests with Welch’s correction. Comparisons involving multiple markers across the two groups were analyzed using two-way ANOVA with multiple comparisons test. For qRT-PCR, *p*-values were additionally corrected using the Benjamini–Hochberg FDR procedure. Statistical significance was defined as * *p* < 0.05, ** *p* < 0.01, *** *p* < 0.001, **** *p* < 0.0001; ns, not significant.

## 3. Results

### 3.1. Tjap1 Is Expressed in Brain Microvascular Endothelial Cells and Localizes Predominantly to the Perinuclear Region

**Figure 1:**
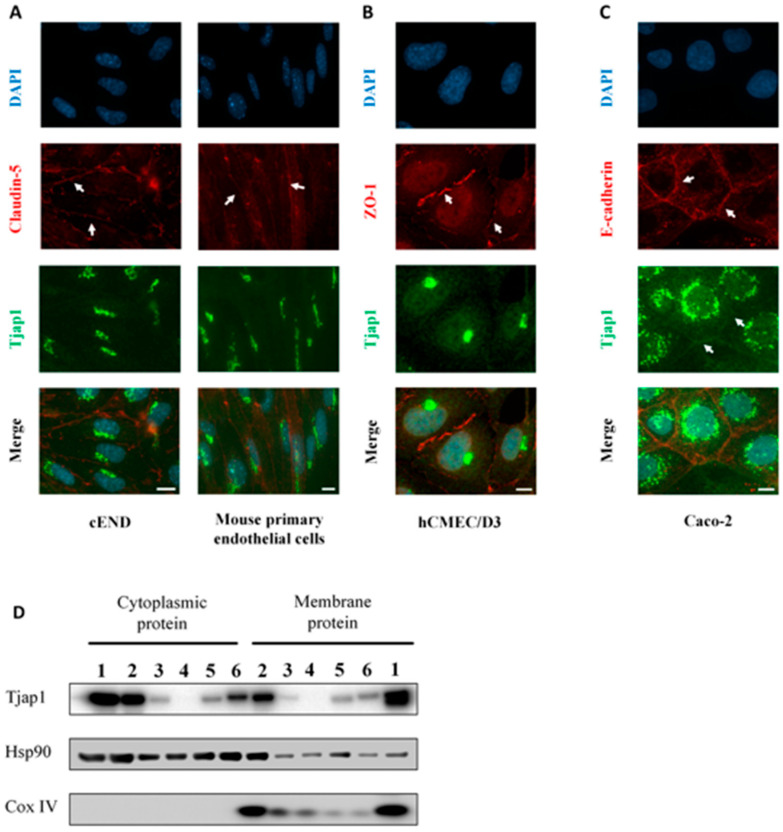
Tjap1 is expressed in brain endothelial cells and localizes predominantly to the perinuclear region. (**A**–**C**) Double immunofluorescence staining of Tjap1 (green) with junctional markers (red) in brain microvascular endothelial cells. (**A**) cEND cells and primary mouse brain endothelial cells co-stained for claudin-5 and Tjap1. Claudin-5 shows a typical continuous staining along cell–cell contacts (arrows), while Tjap1 displays concentrated perinuclear staining without a tight junction (TJ)-associated signal. (**B**) hCMEC/D3 cells co-stained for ZO-1 and Tjap1. ZO-1 exhibits junctional staining at cell borders (arrows), while Tjap1 shows strong perinuclear accumulation without detectable TJ-localization. (**C**) Caco-2 cells co-stained for E-cadherin and Tjap1. E-cadherin displays typical junctional staining at cell–cell contacts (arrows). Tjap1 shows prominent perinuclear staining accompanied by weak diffuse cytoplasmic distribution and weak junctional-associated signal (arrows). Nuclei are stained with DAPI (blue). Merged images are shown in the bottom row. Scale bars = 10 μm. (**D**) Western blot analysis of Tjap1 in cytoplasmic and membrane protein fractions. 1: hCMEC/D3; 2: Caco-2; 3: cEND wild type; 4: cEND Tjap1 knockout; 5: cEND control; 6: primary mouse brain endothelial cells. Hsp90 serves as a cytoplasmic fraction marker; Cox IV serves as a membrane fraction marker.

To characterize the expression and subcellular distribution of Tjap1 in BMECs, we performed double immunofluorescence staining in cEND, hCMEC/D3 cells, and primary mouse BMECs. In cEND cells, claudin-5 exhibited a continuous linear staining pattern along the cell–cell contacts (Figure 1A, arrows), while Tjap1 showed a strong perinuclear signal concentrated in the juxtanuclear region, with weak diffuse cytoplasmic staining and no co-localization with claudin-5 at TJs (Figure 1A). A similar pattern was observed in primary mouse BMECs (Figure 1A). In hCMEC/D3 cells, ZO-1 showed junctional staining at the cell boundaries (Figure 1B, arrows), while Tjap1 showed strong perinuclear accumulation without detectable TJ-associated signal (Figure 1B). Notably, no clear, continuous TJ-associated linear staining pattern for Tjap1 was observed in any of the three cell types, suggesting that Tjap1 is predominantly localized in intracellular compartments rather than at the TJs in BMECs. For comparison, we investigated the immunostaining of Tjap1 in Caco-2 cells, which are known to form well-developed intercellular junctions [28]. The results showed that E-cadherin exhibited a junction-specific staining pattern (Figure 1C, arrows), while Tjap1 showed pronounced perinuclear staining with weak cytoplasmic distribution and a weak junction-associated signal (Figure 1C, arrows). This is consistent with previous reports describing the incorporation of Tjap1 into epithelial TJs [20].

To further investigate the membrane protein association of Tjap1, we performed subcellular fractionation followed by Western blot (Figure 1D). Hsp90 was enriched in the cytoplasmic fraction, and Cox IV was enriched in the membrane fraction, confirming successful fractionation. Tjap1 was detected in both cytosolic and membrane fractions in all cell types examined. Notably, the intensity of Tjap1 in the membrane fraction was stronger in Caco-2, hCMEC/D3, and primary mouse BMECs compared to cEND cells, suggesting that the proportion of membrane-associated Tjap1 varies across cell types with different junctional properties.

### 3.2. Tjap1 Is Predominantly Associated with the cis-Golgi Compartment in the Brain Microvascular Endothelial Cells

**Figure 2:**
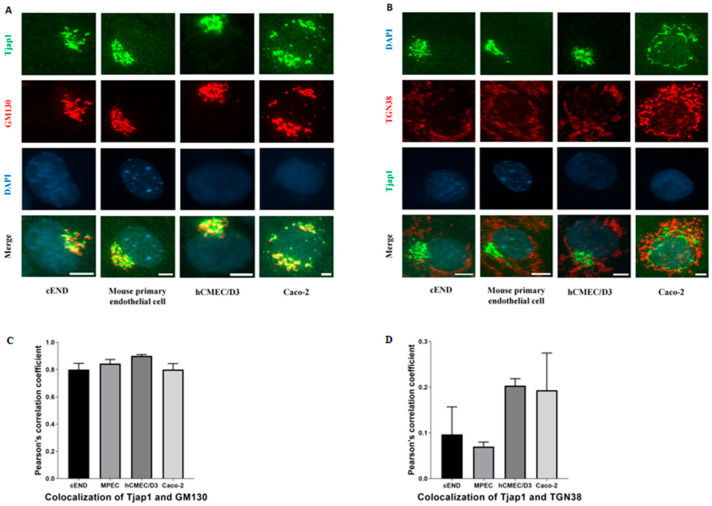
Tjap1 co-localizes with the cis-Golgi marker GM130 but not the trans-Golgi network marker TGN38 in BMECs. (**A**) Double immunofluorescence staining of Tjap1 (green) and the cis-Golgi marker GM130 (red) in Caco-2, cEND, hCMEC/D3, and primary mouse brain endothelial cells. Nuclei were counterstained with DAPI (blue). (**B**) Double immunofluorescence staining of Tjap1 (green) and the trans-Golgi network marker TGN38 (red) in the same four cell types. Scale bars = 5 μm. (**C**) Quantification of the co-localization of Tjap1 and GM130 across various cell types. Pearson correlation coefficients were calculated for cEND, MPEC (primary mouse brain endothelial cells), hCMEC/D3, Caco-2 cells. (**D**) Quantification of Tjap1 and TGN38 co-localization across various cell types. Pearson correlation coefficients were calculated for cEND, MPEC (primary mouse brain endothelial cells), hCMEC/D3, Caco-2 cells. Pearson correlation coefficients were calculated using ImageJ. Data are presented as mean ± SD, *n* = 3.

To define the subcellular compartment underlying the strong perinuclear accumulation of Tjap1 in BMECs, we performed double immunofluorescence staining with established Golgi markers. Co-staining of Tjap1 with the cis-Golgi marker Gm130 showed distinct co-localization in cEND, hCMEC/D3, primary mouse BMECs, and Caco-2 cells. In each cell type, both signals exhibited characteristic perinuclear, ribbon-like Golgi patterns with strong overlap (Figure 2A). In contrast, co-staining of Tjap1 with the trans-Golgi network marker Tgn38 showed only minimal overlap across all four cell types. The Tgn38 staining appeared more scattered and broadly distributed throughout the perinuclear cytoplasm, while Tjap1 remained concentrated in a more compact juxtanuclear region (Figure 2B). A quantitative analysis using Pearson’s correlation coefficient confirmed strong co-localization of Tjap1 and Gm130 in cEND cells (r = 0.80), primary mouse BMECs (r = 0.84), hCMEC/D3 (r = 0.90), and Caco-2 cells (r = 0.80), in contrast to the very low correlations of Tjap1 and Tgn38 observed in cEND cells (r = 0.10), primary mouse BMECs (r = 0.07), hCMEC/D3 (r = 0.20), and Caco-2 cells (r = 0.19) (Figure 2C,D). These results indicate that Tjap1 is preferentially located in the cis-Golgi network rather than the trans-Golgi network in the endothelial and epithelial cells studied.

**Figure 3:**
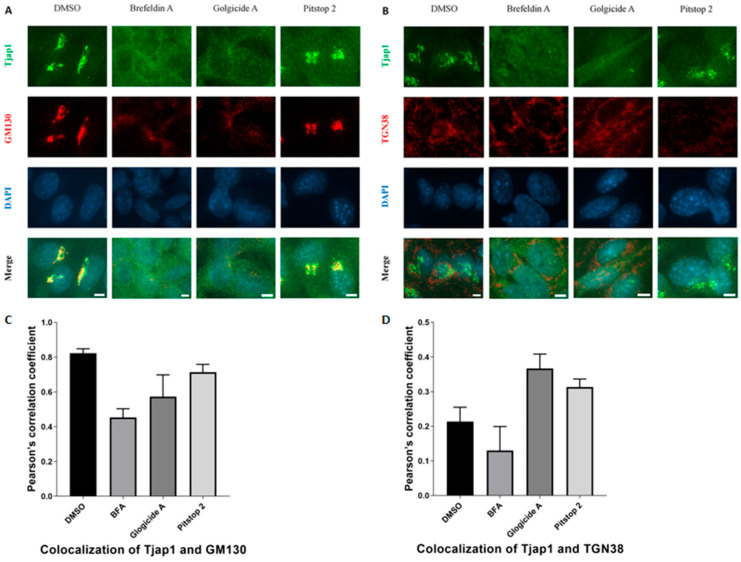
Tjap1 responds to cis-Golgi-disrupting agents in BMECs. (**A**) cEND cells treated with vehicle (DMSO), Brefeldin A (BFA), Golgicide A, or Pitstop 2 and co-stained for GM130 (red) and Tjap1 (green). (**B**) cEND cells treated as in (**A**) and co-stained for TGN38 (red) and Tjap1 (green). Scale bars = 5 μm. (**C**) Quantification of the co-localization of Tjap1 and GM130 in cEND cells after pharmacological treatment. Pearson correlation coefficients were calculated for treatments with DMSO, BFA, Golgicide A, and Pitstop 2. (**D**) Quantification of the co-localization of Tjap1 and TGN38 in cEND cells after pharmacological treatment. Pearson correlation coefficients were calculated for DMSO, BFA, Golgicide A, Pitstop 2. Pearson correlation coefficients were calculated using ImageJ software. Data are presented as mean ± SD, *n* = 3.

To further confirm this cis-Golgi association, we employed pharmacological agents that differentially affect Golgi compartments. In vehicle-treated cEND cells, Tjap1 and Gm130 exhibited their characteristic co-localized perinuclear distribution (Figure 3A, first column). Treatment of cEND cells with BFA resulted in marked redistribution of both signals: the concentrated perinuclear Gm130 signal was replaced by scattered or fragmented structures distributed throughout the perinuclear region, and Tjap1 exhibited a parallel pattern, losing its characteristic juxtanuclear localization and becoming dispersed throughout the cytoplasm (Figure 3A, second column). In the corresponding Tgn38 co-staining, BFA treatment left Tgn38 staining largely unchanged, while Tjap1 lost its compact juxtanuclear pattern and became diffusely dispersed (Figure 3B, second column), demonstrating that Tjap1 responds selectively to cis-Golgi disruption. Similarly, treatment with Golgicide A caused comparable dispersal of both Tjap1 and Gm130 (Figure 3A, third column), whereas Tgn38 staining again remained unaffected despite the loss of the Tjap1 juxtanuclear pattern (Figure 3B, third column). Conversely, treatment with Pitstop 2 did not alter the co-localized perinuclear distribution of Tjap1 and Gm130 (Figure 3A, fourth column), but caused partial disappearance of Tgn38 staining, while Tjap1 retained its compact juxtanuclear pattern (Figure 3B, fourth column). This reciprocal pattern provides strong pharmacological evidence that Tjap1 locates at the cis-Golgi compartment rather than the trans-Golgi network. Quantification using the Pearson’s correlation coefficient supported these observations: The co-localization of Tjap1 and Gm130 in cEND cells decreased significantly upon treatment with BFA (r = 0.45) and Golgicide A (r = 0.57) compared with vehicle controls (r = 0.82), yet remained largely preserved upon treatment with Pitstop 2 (r = 0.71). The correlations between Tjap1 and Tgn38 remained consistently low under all conditions (r = 0.13–0.37), confirming the selective response of Tjap1 to the disruption of cis-Golgi structure (Figure 3C,D). Similar results were observed in hCMEC/D3 cells, primary mouse brain endothelial cells, and Caco-2 cells (Supplementary Figure S1).

### 3.3. Tjap1 Is Recruited Late and Weakly to Cell–Cell Junctions During Endothelial Wound Healing

**Figure 4:**
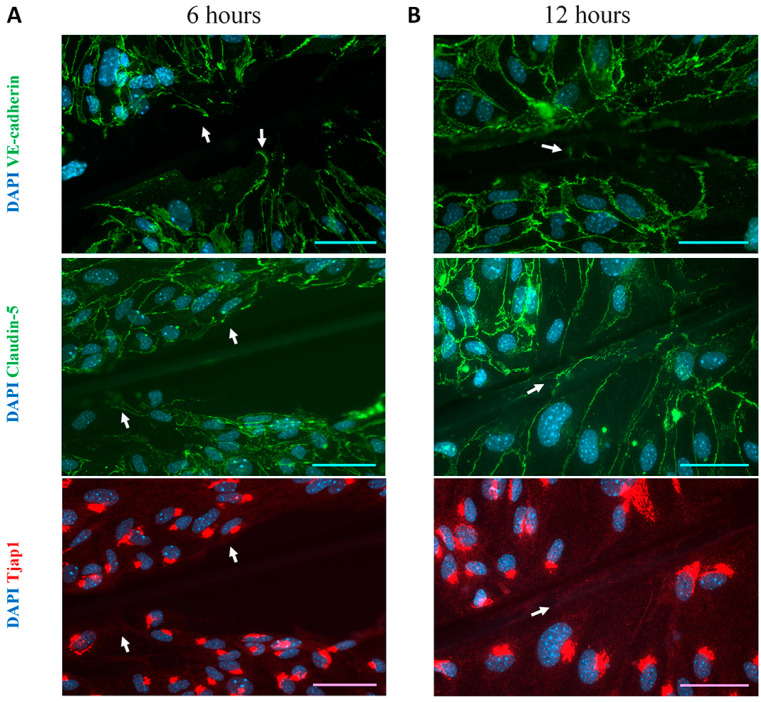
Tjap1 does not accumulate at newly forming cell–cell junctions during endothelial wound healing. Scratch wound healing assays were performed on confluent primary mouse brain endothelial monolayers. Cells were fixed at 6 h (**A**) and 12 h (**B**) post-wounding and stained for VE-cadherin (green, top row), claudin-5 (green, middle row), or Tjap1 (red, bottom row). Nuclei were counterstained with DAPI (blue). (**A**) At 6 h, VE-cadherin and claudin-5 had already accumulated at cell–cell contacts along the wound edge (arrows), whereas Tjap1 remained concentrated in the perinuclear region with no junctional enrichment (arrows). (**B**) By 12 h, VE-cadherin and claudin-5 had formed more continuous junctional strands at the healing site (arrows), while Tjap1 continued to display a cytoplasmic distribution without clear tight junction localization (arrow). Scale bars = 50 μm.

To assess whether Tjap1 participates in the dynamic assembly of endothelial cell–cell junctions, we performed scratch wound healing assays in confluent primary mouse BMECs and examined the temporal recruitment of Tjap1 relative to classical junctional markers. Confluent monolayers were manually scratched with a needle and then cultured for 3, 6, and 12 h. Co-staining for Tjap1 was then performed with either VE-cadherin or claudin-5. Since no significant changes in the staining patterns were observed after 3 h, we focused on the 6 and 12 h time points. At 6 h post-wounding, VE-cadherin and claudin-5 accumulated at cell–cell contacts along the wound edge (Figure 4A, arrows). Twelve hours post-scratch, VE-cadherin formed discrete dots and short linear structures at new adhesion sites, while claudin-5 increasingly organized into continuous, belt-like strands along the recovering wound margins (Figure 4B, arrows). The observed recruitment dynamics are consistent with the established principle that cadherin-based adherens junctions form early during cell–cell contact and are required for the subsequent formation of TJs [29,30].

Conversely, Tjap1 did not accumulate at the earliest cell junctions. In the initial stages, Tjap1 staining was mainly localized around the nucleus, with only diffuse cytoplasmic staining and no distinct accumulation of Tjap1 at newly formed wound edges (Figure 4A, arrows). In later stages of wound healing, when VE-cadherin and claudin-5 had formed cell junction belts, Tjap1 also appeared at the healing site; however, compared to the typical clear membrane-associated pattern of VE-cadherin and claudin-5, its staining showed no TJ signal and was more strongly directed towards cytoplasmic distribution (Figure 4B, arrow).

### 3.4. RNAscope In Situ Hybridization Reveals a Predominantly Perinuclear Distribution of Tjap1 mRNA in Brain Microvascular Endothelial Cells

**Figure 5:**
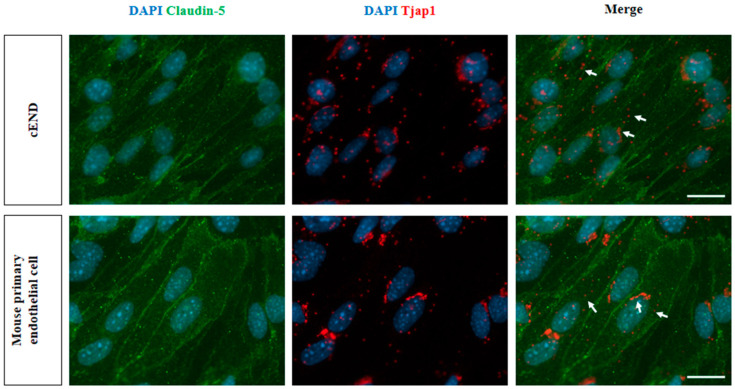
Tjap1 mRNA localizes predominantly to the perinuclear region and does not co-localize with tight junctions in BMECs. RNAscope in situ hybridization for Tjap1 mRNA combined with immunofluorescence staining for claudin-5 in cEND cells (top row) and primary mouse brain endothelial cells (bottom row). Left column: DAPI (blue) and claudin-5 (green), showing continuous junctional strands at cell–cell borders. Middle column: DAPI (blue) and Tjap1 mRNA (red), detected as discrete punctate signals concentrated in the perinuclear region. Right column: merged images showing that Tjap1 mRNA puncta (arrows) are spatially separate from claudin-5 junctional strands. Scale bars = 20 μm.

To examine the subcellular distribution of Tjap1 mRNA, we performed RNAscope in situ hybridization combined with immunofluorescence staining of claudin-5 in cEND cells and primary mouse BMECs. The positive and negative controls included in every RNAscope experiment confirmed RNA integrity as well as the absence of nonspecific hybridization. Claudin-5 staining showed cell–cell junctions as continuous linear strands along cell borders in both cell types (Figure 5, left column). Tjap1 mRNA was detected as discrete punctate signals (Figure 5, middle column). In both cell types, most Tjap1 mRNA puncta formed perinuclear clusters in close proximity to the nucleus, while additional, scattered signals were distributed throughout the cytoplasm. We also observed that the Tjap1 mRNA puncta were spatially separated from the claudin-5-positive cell junctions and showed no co-localization with TJ structures at the cell membrane (Figure 5, right column, arrows).

### 3.5. Tjap1 Knockout Induces Golgi Fragmentation

**Figure 6:**
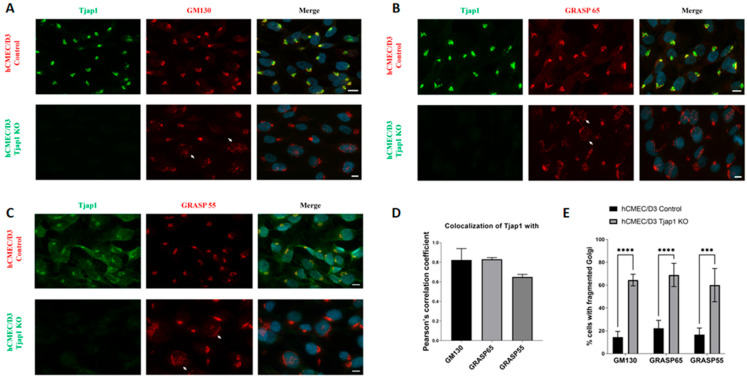
Tjap1 knockout induces Golgi fragmentation in BMECs. (**A**–**C**) Double immunofluorescence staining of Tjap1 with the cis-Golgi markers Gm130 (**A**) and Grasp65 (**B**), and the medial/trans-Golgi marker Grasp55 (**C**) in control and Tjap1 knockout hCMEC/D3 cells. In Tjap1 knockout cells, Tjap1 staining is abolished, confirming efficient knockout. Gm130, Grasp65, and Grasp55 all exhibit dispersed punctate staining patterns in Tjap1 knockout cells (arrows), replacing the compact perinuclear ribbon morphology observed in controls. Scale bar = 10 µm. (**D**) Quantification of the co-localization of Tjap1 with GM130, GRASP65, and GRASP55 in control hCMEC/D3 cells. Pearson correlation coefficients were calculated for Tjap1/GM130, Tjap1/GRASP65, and Tjap1/GRASP55. Pearson correlation coefficients were calculated using ImageJ software. Data are presented as mean ± SD, *n* = 3. (**E**) Quantification of Golgi morphology in control and Tjap1 KO hCMEC/D3 cells. Golgi morphology was classified for GM130, GRASP65, and GRASP55 based on blinded assessment of 30 cells per sample across three biological replicates. Statistical significance was determined by two-way ANOVA with multiple comparisons. Data are presented as mean ± SD. *** *p* < 0.001; **** *p* < 0.0001.

To investigate whether Tjap1 contributes to Golgi organization in BMECs, we first examined its relationship with Golgi matrix proteins by double immunofluorescence in hCMEC/D3 cells. Quantification of co-localization in control hCMEC/D3 cells revealed a strong overlap of Tjap1 with Gm130 (Pearson r = 0.82) and Grasp65 (r = 0.83), as well as a moderate overlap with Grasp55 (r = 0.65) (Figure 6D). These results demonstrated that Tjap1 co-localized with Grasp65 and Gm130 and partially co-localized with Grasp55 (Figure 6A–C), consistent with its cis-Golgi localization.

We next investigated whether Tjap1 is required for the structural integrity of the Golgi apparatus. In hCMEC/D3 Tjap1 knockout cells, the Tjap1 signal was absent, confirming a successful knockout (Figure 6A–C, lower left panels). Notably, Gm130 staining in Tjap1 knockout cells no longer showed the organized, compact ribbon morphology observed in controls, but instead revealed scattered, punctate structures distributed in the perinuclear and cytoplasmic areas (Figure 6A, arrows). A similar fragmentation phenotype was observed for Grasp65 (Figure 6B, arrows) and Grasp55 (Figure 6C, arrows), both of which showed scattered, punctate patterns instead of the characteristic compact Golgi ribbon. These results indicate that Tjap1 knockout disrupts the structure of both cis-Golgi (Gm130, Grasp65) and medial/trans-Golgi (Grasp55) compartments in hCMEC/D3 cells. Quantification of Golgi morphology revealed a significant increase in cells with fragmented Golgi structures for Gm130, Grasp65, and Grasp55 in Tjap1 knockout hCMEC/D3 cells compared to controls (Figure 6E). Comparable Golgi fragmentation phenotypes were observed in cEND cells with Tjap1 knockout (Supplementary Figure S2), indicating that the necessity of Tjap1 for maintaining Golgi integrity is consistent across all BMEC lines.

As an initial exploratory observation, we performed glycoprotein gel staining on two different Tjap1 knockout cell lines, as well as their corresponding control cell lines. In the cEND Tjap1 knockout cells, a distinct band was observed at approximately 55–70 kDa, which was absent in the controls; conversely, no obvious differences could be detected in the hCMEC/D3 cells (Supplementary Figure S3). The identity of this band, as well as the observed species difference, were not further investigated in the present study and require further characterization.

### 3.6. Tjap1 Knockout Alters Expression of Drug Transporters and Cellular Receptors in Brain Microvascular Endothelial Cells

**Figure 7:**
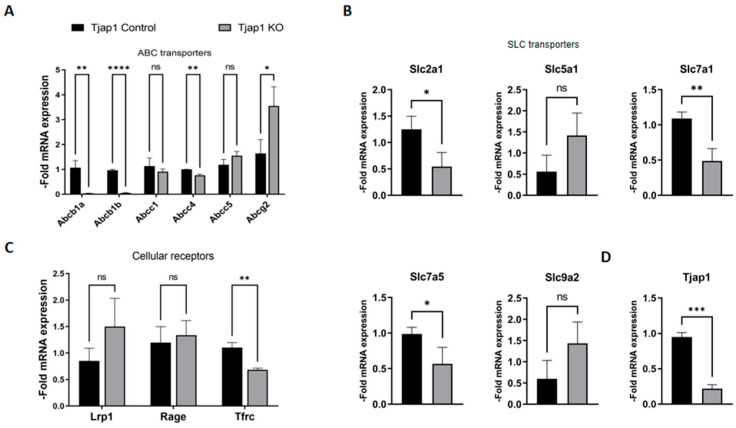
Tjap1 knockout alters mRNA expression of ABC efflux transporters, SLC transporters, and cellular receptors in microvascular brain endothelial cells. Quantitative real-time PCR analysis of blood–brain barrier-associated transporter and receptor mRNA expression in Tjap1 Control and Tjap1 KO cEND cells. Target gene expression of ABC transporters (**A**), SLC transporters (**B**), and cellular receptors (**C**) was normalized to the endogenous control and presented as fold change relative to control (mean ± SD; *n* = 3). Statistical analysis was performed using unpaired *t*-tests with Welch’s correction. * *p* < 0.05; ** *p* < 0.01; **** *p* < 0.0001; ns, not significant. (**A**) Abcb1a, P-Glycoprotein, ATP binding cassette subfamily B member 1A; Abcb1b, P-Glycoprotein, ATP binding cassette subfamily B member 1; Abcc1, Mrp1, Multidrug resistance associated protein 1; Abcc4, Mrp4, Multidrug resistance associated protein 4; Abcc5, ATP binding cassette subfamily C member 5; Abcg2, BCRP, Breast cancer resistance protein. (**B**) Slc2a1, Solute carrier family 2 member 1, Glut1, Glucose transporter type 1; Slc5a1, Solute carrier family 5 member 1, Sglt1, Sodium-glucose cotransporter 1; Slc7a1, Solute carrier family 7 member 1, Cat1, Cationic amino acid transporter 1; Slc7a5, Solute carrier family 7 member 5, Lat1, L-type amino acid transporter 1; Slc9a2, Solute carrier family 9 member 2, Nhe2, Sodium-hydrogen exchanger 2. (**C**) Lrp1, Low-density lipoprotein receptor-related protein 1; Rage, Receptor for Advanced Glycosylation End Products; Tfrc, Transferrin receptor. (**D**) Quantitative real-time PCR validation of Tjap1 mRNA expression in Tjap1 knockout and control cEND cells (mean ± SD; *n* = 3). Statistical analysis was performed using unpaired *t*-tests with Welch’s correction. *** *p* < 0.001.

To investigate whether Tjap1 regulates the expression of BBB-associated transporters, we analyzed the mRNA levels of key efflux pumps, solute carrier transporters, and cellular receptors in cEND Tjap1 knockout and control cells by quantitative real-time PCR. Among the ABC efflux transporters (Figure 7A), Tjap1 knockout resulted in a marked downregulation of Abcb1a (*p* < 0.01) and Abcb1b (*p* < 0.0001), two genes encoding P-glycoprotein at the BBB. Abcc4 was also significantly reduced in Tjap1 knockout cells (*p* < 0.01). No significant changes were detected for Abcc1 or Abcc5. In contrast, Abcg2 was significantly upregulated in Tjap1 knockout cells (*p* < 0.05). Among the SLC transporters (Figure 7B), Slc2a1 (*p* < 0.05), Slc7a1 (*p* < 0.01), and Slc7a5 (*p* < 0.05) were all significantly downregulated in Tjap1 knockout cells. In contrast, Slc5a1 (Sglt1, sodium-glucose cotransporter 1) and Slc9a2 (Nhe2, sodium-hydrogen exchanger 2) both showed non-significant trends toward increased expression in Tjap1 knockout cells. Among the cellular receptors (Figure 7C), Tfrc was significantly decreased in Tjap1 knockout cells (*p* < 0.01), while Lrp1 and the receptor for advanced glycation end products (Rage) showed non-significant increases. Meanwhile, we also observed that Tjap1 knockdown in cEND cells showed broadly consistent expression trends for the majority of the drug transporters and receptors (Supplementary Figure S4).

**Figure 8:**
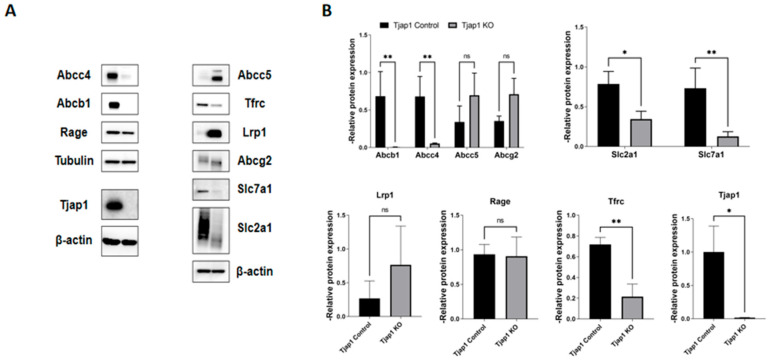
Tjap1 knockout alters protein expression of ABC efflux transporters, solute carrier transporters, and cellular receptors in brain microvascular endothelial cells. (**A**) Representative Western blot images showing the protein expression of Abcc4, Abcb1, Rage, Tjap1, Abcc5, Tfrc, Lrp1, Abcg2, Slc7a1, Slc2a1, and β-actin in Tjap1 Control and Tjap1 KO cEND cells. β-actin or tubulin served as loading controls. (**B**) Quantification of relative protein expression levels, normalized to the respective loading control. The grayscale values of the bands were quantitatively analyzed using ImageJ software. Statistical significance was determined by unpaired *t*-tests with Welch’s correction. Data are presented as mean ± SD (*n* = 3). * *p* < 0.05, ** *p* < 0.01; ns, not significant.

We further investigated the effect of Tjap1 knockout on the protein expression levels of representative ABC efflux transporters, SLC transporters, and cellular receptors (Figure 8A,B). Consistent with the mRNA results, Abcb1 (*p* < 0.01) and Abcc4 (*p* < 0.01) protein levels were significantly reduced in Tjap1 KO cEND cells. The protein levels of Abcc5 and Abcg2 did not change significantly, although both exhibited a non-significant upward trend. Among the SLC transporters, the protein levels of both Slc2a1 (*p* < 0.05) and Slc7a1 (*p* < 0.01) were significantly downregulated in Tjap1 KO cells, consistent with their mRNA profiles. The Tfrc protein was also markedly reduced (*p* < 0.01), consistent with its mRNA downregulation. The protein levels of Lrp1 and Rage did not differ significantly between the two groups, although Lrp1 showed a non-significant upward trend. Tjap1 protein was undetectable in the Tjap1 knockout cEND cells (*p* < 0.05), confirming successful knockout at the protein level. Taken together, these data at the protein-level largely validate the mRNA findings and suggest that Tjap1 knockout could lead to comprehensive changes in the expression of BBB-associated transporters and receptors.

## 4. Discussion

In this study, we characterized the expression and subcellular localization of Tjap1 in BMECs, and investigated its role in the integrity of Golgi apparatus, as well as in the expression of BBB-associated drug transporters and receptors. We found that Tjap1 in BMECs is predominantly localized within the cis-Golgi compartment, rather than at TJs or in the trans-Golgi network, as previously reported for other cell types. Silencing of Tjap1 resulted in pronounced fragmentation of the Golgi apparatus and altered glycoprotein profiles, suggesting that Tjap1 is essential for the structural integrity of Golgi apparatus in BMECs. In addition, Tjap1 knockout significantly altered the mRNA and protein expression of key BBB-associated drug transporters as well as cellular receptors. Taken together, these results indicate that Tjap1 is required for Golgi architecture and for the expression of transporters and receptors that contribute to the barrier properties of the BBB. This opens up the possibility of specifically targeting Tjap1 to modulate drug transport across the BBB.

Previous studies reported conflicting subcellular localizations for Tjap1 depending on the cell type examined. Kawabe et al. first identified Tjap1 as a novel peripheral membrane protein at TJs in MTD-1A epithelial cells, noting that Tjap1 was incorporated into TJs at a late stage after claudin-based junctional strands had already formed [19]. Subsequently, Tamaki et al. identified Tjap1 as an Arf6-binding protein that predominantly localized to the trans-Golgi complex in NIH3T3 fibroblasts [20]. Our results revealed that in all three BMECs types examined—cEND, hCMEC/D3, and primary mouse BMECs—Tjap1 displayed a strong perinuclear signal with weak diffuse cytoplasmic staining, consistent with an intracellular organelle-associated distribution. Importantly, Tjap1 did not exhibit the continuous linear staining pattern characteristic of TJs such as claudin-5 and ZO-1. In Caco-2 epithelial cells; however, Tjap1 displayed predominantly perinuclear staining accompanied by weak cytoplasmic and TJ-associated signals, whereas BMECs showed no detectable TJ localization, suggesting that the recruitment of Tjap1 to cell–cell junctions is cell-type dependent and may correlate with the level of TJs development. Consistent with this notion, subcellular fractionation revealed that Tjap1 was present in both cytosolic and membrane fractions, with the membrane-associated Tjap1 signal being notably stronger in Caco-2, hCMEC/D3, and primary mouse BMECs compared to cEND cells. These observations suggest that while the predominant pool of Tjap1 resides at the perinuclear region across all BMEC models examined, a minor fraction may be recruited to the plasma membrane in cells with more developed intercellular junctions. Notably, Caco-2 epithelial cells form a particularly robust paracellular barrier with high expression of occludin and epithelial claudins (claudin-1, -3, -4, and -7), whereas brain endothelial cells are characterized by a distinct claudin profile dominated by claudin-5 and low expression of epithelial claudins [31]. Moreover, among immortalized brain endothelial cell lines, hCMEC/D3 cells retain relatively higher expression of occludin and claudin-5 compared to other lines such as GP8 and RBE4, yet their levels remain markedly lower than those of primary mouse BMECs [31,32].

Co-localization analysis with Golgi markers demonstrated that Tjap1 extensively overlapped with the cis-Golgi marker Gm130 but showed almost no co-localization with the trans-Golgi marker Tgn38. To define the Golgi sub-compartment associated with Tjap1, we employed three pharmacological agents with distinct mechanisms of action targeting different Golgi compartments. BFA and Golgicide A both inhibit cis-Golgi function through GBF1, a cis-Golgi-localized ArfGEF—BFA blocks ER-to-Golgi trafficking leading to Golgi absorption into the ER, while Golgicide A causes rapid dissociation of COPI coat proteins and subsequent Golgi fragmentation [33,34]. In contrast, Pitstop 2 primarily impacts clathrin-dependent processes at the TGN and plasma membrane [35]. The concordant response of Tjap1 with Gm130 to cis-Golgi-targeting agents (BFA and Golgicide A) and its insensitivity to Pitstop 2, across all cell types examined, provides strong pharmacological evidence that Tjap1 resides at the cis-Golgi compartment. This finding contrasts with the previously reported trans-Golgi localization in NIH3T3 fibroblasts [20], thereby underscoring that localization of Tjap1 is cell-type specific. Consistent with the cis-Golgi protein localization, our RNAscope in situ hybridization analysis revealed that Tjap1 mRNA was predominantly distributed as perinuclear puncta and scattered cytoplasmic signals, with no appreciable co-localization with claudin-5 at cell–cell junctions. This perinuclear mRNA distribution is in agreement with the protein localizing to the Golgi apparatus. Furthermore, our wound healing experiments showed that Tjap1 did not accumulate at the earliest junctions and only appeared weakly at cell–cell contacts at later stages, with a cytoplasmic distribution rather than the sharp membrane-associated pattern seen for VE-cadherin and claudin-5, further supporting its primary role as a Golgi-resident protein rather than a constitutive TJ component. Given that Tjap1 was originally identified as an Arf6-binding protein involved in Golgi-to-membrane trafficking [20], Tjap1 may participate in vesicle transport from the Golgi apparatus to the plasma membrane near junction sites under certain conditions, although this remains to be experimentally verified.

The structural integrity of the Golgi ribbon is maintained by matrix proteins that serve distinct but complementary roles. Gm130 is a cis-Golgi matrix protein that, together with p115 and Grasp65, tethers ER-derived carriers into Golgi stacks [36]. And in the absence of Gm130, this process is impaired, leading to shortening of cisternae and breakdown of the Golgi ribbon [37]. Grasp65 and Grasp55 are Golgi reassembly stacking proteins localized to the cis and medial-trans cisternae, respectively, where they form trans-oligomers across adjacent cisternae to hold them together in a stack, and depletion of either GRASP reduces the number of cisternae per stack, whereas simultaneous knockout of both disperses the entire stack into single cisternae and tubulovesicular structures [38,39]. In this study, silencing Tjap1 in cEND and hCMEC/D3 cells resulted in pronounced Golgi fragmentation. In control cells, Gm130, Grasp65, and Grasp55 staining revealed compact perinuclear Golgi ribbon morphology, whereas Tjap1 knockout cells displayed dispersed punctate structures throughout the cytoplasm, indicating disruption of both cis-Golgi (Gm130, Grasp65) and medial/trans-Golgi (Grasp55) compartments. In Tjap1-knockout hCMEC/D3 cells, the proportion of cells with Golgi fragmentation was significantly increased for all three Golgi structural markers. This phenotype is consistent with the Golgi fragmentation observed upon Tjap1 silencing in NIH3T3 fibroblasts [20], indicating that Tjap1 contributes to the maintenance of Golgi ribbon integrity across multiple cell types. Since proper Golgi ribbon organization facilitates uniform distribution of glycosylation enzymes across stacks to ensure accurate glycan processing [40,41], we performed an exploratory glycoprotein gel staining of Tjap1 knockout and control cell lines. A differential band was detected in cEND Tjap1 knockout cells; however, this band was not observed in hCMEC/D3 knockout cells. As this band has not yet been identified, its biological significance remains to be elucidated in future studies.

Beyond its role in Golgi integrity, a key finding of the present study is that Tjap1 knockout significantly altered the mRNA and protein expression of multiple BBB-associated transporters and receptors. Among the ABC efflux transporters, Abcb1a and Abcb1b mRNA were markedly downregulated, both genes encoding P-glycoprotein, the major drug efflux pump at the BBB that actively restricts the brain penetration of a wide range of structurally diverse lipophilic drugs, including chemotherapeutic agents, antiretrovirals, and immunosuppressants [3]. Abcc4 mRNA was also significantly reduced. Mrp4 is a major efflux transporter expressed at the BBB, localized primarily to the luminal membrane of BMECs, where it limits the brain distribution of organic anions including the anticancer drug topotecan, the antiviral drug adefovir, the antifolate methotrexate, and the anti-influenza agent oseltamivir [42,43]. Western blot analysis further confirmed that the protein levels of Abcb1 and Abcc4 were significantly reduced in Tjap1 knockout cEND cells. The decreased expression of Abcb1a/b and Abcc4 indicates that Tjap1 is required for the normal expression of the major efflux transporters that restrict CNS drug penetration. In contrast, Abcg2 (Bcrp) mRNA was significantly upregulated, whereas Abcg2 protein level in Tjap1 knockout cells showed no significant change. The discrepancy observed for Abcg2 between mRNA and protein levels may reflect post-transcriptional and post-translational regulatory mechanisms. The mRNA and protein levels for the same gene often correlate imperfectly, driven by processes such as microRNA-mediated regulation, translation efficiency, and ubiquitin-mediated proteasomal turnover, which can uncouple steady-state protein levels from transcript abundance [44]. Bcrp is expressed at high levels on the luminal membrane of BMECs, where it functions alongside P-glycoprotein as a dominant drug efflux barrier, actively extruding a broad range of substrates including mitoxantrone, topotecan, methotrexate, and it also interacts with tyrosine kinase inhibitors such as imatinib and gefitinib, influencing their CNS penetration [45,46].

Among the SLC transporters, Slc2a1 (Glut1), Slc7a1 (Cat1), and Slc7a5 (Lat1) mRNA were all significantly downregulated in cEND Tjap1 knockout cells. The Western blot showed that Slc2a1 and Slc7a1 were also significantly reduced in Tjap1 knockout cEND cells, consistent with their mRNA downregulation. These transporters are critical for CNS nutrient homeostasis and have also been exploited for prodrug delivery strategies: Lat1 mediates the blood-to-brain transport of large neutral amino acids including L-DOPA and several amino acid-mimetic drugs such as melphalan and gabapentin [47]. Glut1 has been explored as a target for glucose-conjugated drug delivery to the brain, whereby glucose moieties are attached to therapeutics to facilitate their transport across the BBB via the Glut1 transporter [48]. Cat1 delivers cationic amino acids essential for nitric oxide synthesis and polyamine metabolism in the CNS [49]. The coordinated downregulation of all three facilitative nutrient transporters suggests that Tjap1 is involved in maintaining the nutrient delivery capacity of the BBB. Interestingly, the sodium-dependent transporters Sglt1 and Nhe2 showed an upward trend in Tjap1 knockout cells, although these changes did not reach statistical significance. Under normal physiological conditions, these transporters are expressed at low levels in BMECs compared to their expression in the intestine and kidney [50,51]. Sglt1 has been demonstrated in the luminal membrane of brain capillary endothelial cells at low levels under basal conditions, but its expression is markedly upregulated following cerebral ischemia and reperfusion injury [52]. Nhe2 is also expressed in the luminal membrane of BBB endothelial cells and Nhe2 activity in BMECs is stimulated by ischemic factors including hypoxia, aglycemia, and arginine vasopressin, and Nhe2 inhibition reduces brain edema following experimental stroke [53]. The upward trend of these sodium-dependent transporters in Tjap1 knockout cells is therefore of potential pathological and pharmacological interest.

Among the cellular receptors, Tfrc mRNA and protein expression were significantly decreased in Tjap1 knockout cEND cells. The downregulation of Tfrc is particularly relevant given its central role in brain iron homeostasis and the growing clinical interest in transferrin receptor-targeted strategies for brain drug delivery, including anti-TfR antibody–drug conjugates and nanoparticle-based platforms designed to exploit receptor-mediated transcytosis across the BBB [54].

Golgi fragmentation has been consistently observed as a pathological feature in Alzheimer’s disease, Parkinson’s disease, and other neurodegenerative conditions [55,56]. In Alzheimer’s disease, aberrant phosphorylation of Grasp65 by Cdk5 disrupts Golgi stacking, leading to enhanced amyloid precursor protein trafficking and increased amyloid-β production [57]. While these studies have focused primarily on neuronal Golgi pathology, the functional consequences of Golgi fragmentation in BMECs remain largely unexplored, therefore our findings add a new dimension by showing that Tjap1 is involved in regulating Golgi stacking and the expression of drug transporters and receptors in BMECs. Whether Tjap1 expression or Golgi integrity is compromised in disease-associated brain endothelium, and whether such changes contribute to the altered drug penetration patterns observed in neurological disorders, warrants further investigation.

The Golgi apparatus contributes to the establishment and maintenance of cell polarity by organizing the polarized membrane trafficking of newly synthesized proteins toward specific surface domains. This function relies in part on scaffolding structures of the cis-Golgi matrix such as GM130 and GRASP65, which regulate Golgi structure, vesicle tethering, and polarity-associated signaling pathways [58,59]. This is particularly relevant for BMECs, which exhibit pronounced apicobasal polarization: their luminal and abluminal membranes differ in lipid and glycoprotein composition, and key transporters and receptors display an asymmetric domain localization that underpins the directional barrier and transport functions of the BBB [60]. Similarly, polarized sorting at the Golgi governs apical–basolateral cargo segregation in epithelial cells, a process in which newly synthesized membrane proteins are differentially delivered to either apical or basolateral domain s [58]. Our study demonstrates that Tjap1 knockout disrupts Golgi architecture, affecting both the cis-Golgi and the medial/trans-Golgi regions, and is accompanied by altered expression of several transporters and receptors that are normally polarized in BMECs. This supports the hypothesis that Tjap1 contributes to the Golgi-dependent maintenance of apicobasal polarity in the brain endothelium. However, the mechanisms by which Tjap1 regulates Golgi organization and polarized cargo processing remain to be elucidated and require further investigation.

Moreover, Tjap1 could influence vesicular trafficking and secretion in BMECs as well as epithelial cells. Tamaki et al. originally identified Tjap1 as an interaction partner of the small GTPase Arf6 [20], a regulator of vesicular transport between the Golgi apparatus, endosomes, and the plasma membrane, as well as of actin remodeling at sites of cargo internalization and delivery [61,62]. Although the primary localization of Tjap1 in BMECs is at the cis-Golgi rather than the trans-Golgi structure, it is nonetheless reasonable to assume that a disruption of cis-Golgi architecture would slow down or misdirect the transit of newly synthesized membrane proteins through the secretory pathway. Direct evidence of Tjap1-dependent vesicular trafficking was beyond the scope of the present study and represents an important avenue for future research.

There are several limitations to the present study. First, our experiments were performed exclusively in vitro using cell lines and primary cell cultures, and in vivo validation of Tjap1 function at the BBB in animal models is needed. Second, while we demonstrated both Golgi fragmentation and transporter expression changes upon Tjap1 knockout, whether these two phenotypes are causally linked or represent independent consequences of Tjap1 loss remains to be determined. Third, the molecular mechanism by which Tjap1 maintains Golgi integrity, and the pathways through which Tjap1 regulates transporter expression, remain to be elucidated. Fourth, although we were able to demonstrate parallel changes at both the mRNA and protein levels for representative transporters and receptors, no functional assays regarding drug transport, efflux, or barrier permeability were conducted. Whether the observed changes in expression actually translate into altered drug permeability across the BBB remains a subject for future investigation. Fifth, no rescue experiments involving Tjap1 re-expression in knockout cells were performed; however, such experiments are necessary to confirm the specificity of the phenotype in future studies. Sixth, the experimental design effectively mitigates the risk of off-target effects in several aspects: the Tjap1 knockout cell lines were generated using a commercially available double-nickase plasmid, which significantly reduces off-target activity compared to wild type Cas9; furthermore, the Golgi fragmentation phenotype was consistently observed in two independently generated knockout cell lines derived from different species (cEND and hCMEC/D3); finally, largely consistent trends regarding transporter expression were also observed in an independent shRNA knockdown cell line. Nevertheless, a formal rescue via Tjap1 re-expression remains the gold standard and will constitute the focus of a future study.

## 5. Conclusions

In conclusion, our study identifies Tjap1 as a cis-Golgi-associated protein in BMECs and shows that it is required for Golgi structural integrity in vitro. Loss of Tjap1 is accompanied by Golgi fragmentation, and significant alterations in the expression of efflux pumps, nutrient transporters, and cellular receptors that contribute to BBB function and CNS drug delivery. These results identify Tjap1 as a potential regulator of the BBB transporter profile and suggest that Tjap1 warrants further investigation as a target for modulating drug transport across the BBB.

## Data Availability

The authors confirm that the data supporting the findings of this study are available within the article and its Supplementary Materials. Raw data are available from the corresponding author upon reasonable request.

## Supplementary Materials

The following supporting information can be downloaded at: https://www.mdpi.com/article/10.3390/pharmaceutics18060665/s1, Figure S1. Tjap1 responds to cis-Golgi-disrupting agents in hCMEC/D3, primary mouse brain endothelial cells, and Caco-2 cells. Figure S2. Tjap1 knockout induces Golgi fragmentation in cEND cells. Figure S3. Glycoprotein gel staining in Tjap1 knockout BMECs. Figure S4. mRNA expression of drug transporters and cellular receptors in cEND Tjap1 knockdown cells.

## Abbreviations

The following abbreviations are used in this manuscript:

ABC	ATP-binding cassette
Abcb1a/b	ATP-binding cassette subfamily B member 1A/1B
Abcc1	ATP-binding cassette subfamily C member 1
Abcc4	ATP-binding cassette subfamily C member 4
Abcc5	ATP-binding cassette subfamily C member 5
Abcg2	ATP-binding cassette subfamily G member 2
Arf6	ADP-ribosylation factor 6
BBB	Blood–brain barrier
BFA	Brefeldin A
BMECs	Brain microvascular endothelial cells
CGN	cis-Golgi network
CNS	Central nervous system
COPI	Coat protein complex I
ER	Endoplasmic reticulum
GM130	Golgi matrix protein 130
GRASP55	Golgi reassembly stacking protein 55
GRASP65	Golgi reassembly stacking protein 65
hDlg	Human discs large tumor suppressor protein
JAMs	Junctional adhesion molecules
Lrp1	Low-density lipoprotein receptor-related protein 1
Nhe2	Sodium–hydrogen exchanger 2
Pilt	Protein incorporated later into tight junctions
Rage	Receptor for advanced glycation end products
Sglt1	Sodium–glucose cotransporter 1
Tfrc	Transferrin receptor
TGN	trans-Golgi network
Tjap1	Tight junction-associated protein 1
TJs	Tight junctions
TLCK	Tosyl-L-lysyl-chloromethane hydrochloride
ZO-1/2/3	Zonula occludens-1/2/3
