# Supplementary material for: Tjap1/Pilt Is a cis-Golgi-Associated Protein Required for Golgi Integrity and Normal Drug Transporter Expression in Brain Microvascular Endothelial Cells In Vitro
Source: Pharmaceutics. 2026 May 28;18(6):665. doi: 10.3390/pharmaceutics18060665 (PMC13305319; doi:10.3390/pharmaceutics18060665)
Supplement: Supplementary file 1 [file pharmaceutics-18-00665-s001.zip › pharmaceutics-4252686-supplementary.pdf]

Article

Tjap1/Pilt Is a cis-Golgi-Associated Protein Required for Golgi Integrity and Normal Drug Transporter Expression in Brain Microvascular Endothelial Cells in Vitro

Junqiao Mi[1,2], Annabelle Schoder[1], Aili Sun[1], Patrick Meybohm[1], Malgorzata Burek[1*]

[1] Department of Anesthesiology, Intensive Care, Emergency and Pain Medicine, University Hospital Würzburg, 97080 Würzburg, Germany
[2] Graduate School of Life Sciences, Julius-Maximilians-Universität Würzburg, 97074 Würzburg, Germany
[*] Correspondence: burek_m@ukw.de

Supplementary Materials

Figure S1

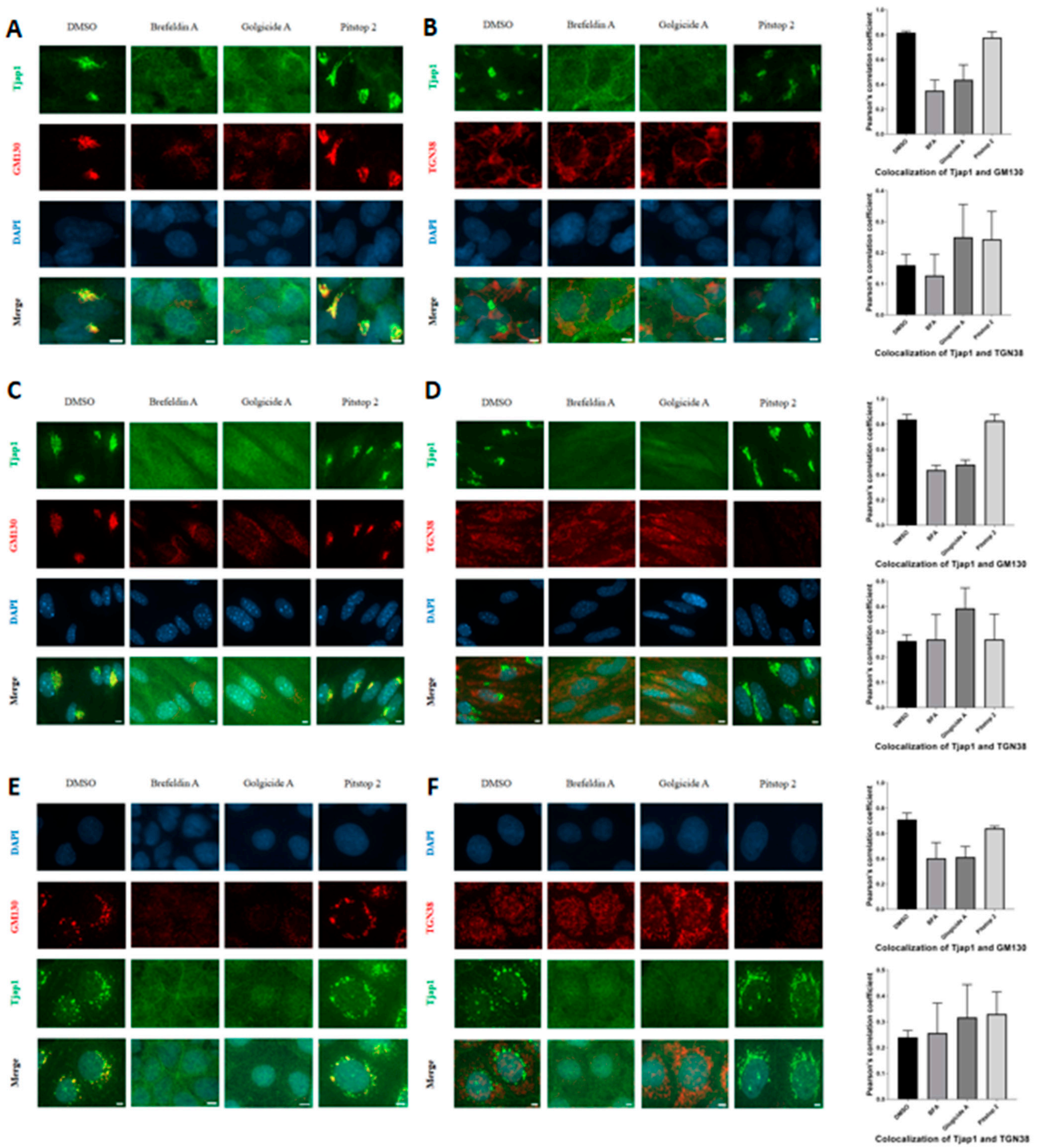

Figure S1. Tjap1 responds to cis-Golgi-disrupting agents in hCMEC/D3, primary mouse brain endothelial cells, and Caco-2 cells. (**A, B**) hCMEC/D3 cells treated with vehicle (DMSO), Brefeldin A (BFA), Golgicide A, or Pitstop 2 and co-stained for GM130 (red) and Tjap1 (green) (**A**) or TGN38 (red) and Tjap1 (green) (**B**). (**C, D**) Primary mouse brain endothelial cells treated as above and co-stained for GM130 (red) and Tjap1 (green) (**C**) or TGN38 (red) and Tjap1 (green) (**D**). (**E, F**) Caco-2 cells treated as above and co-stained for GM130 (red) and Tjap1 (green) (**E**) or TGN38 (red) and Tjap1 (green) (**F**). Nuclei were counterstained with DAPI (blue). In all cell types, BFA and Golgicide A caused dispersal of the compact perinuclear Tjap1 and GM130 signals, consistent with cis-Golgi disruption (see also Figure. 3A, B for cEND cells). Scale bars = 5 µm;

Figure S2

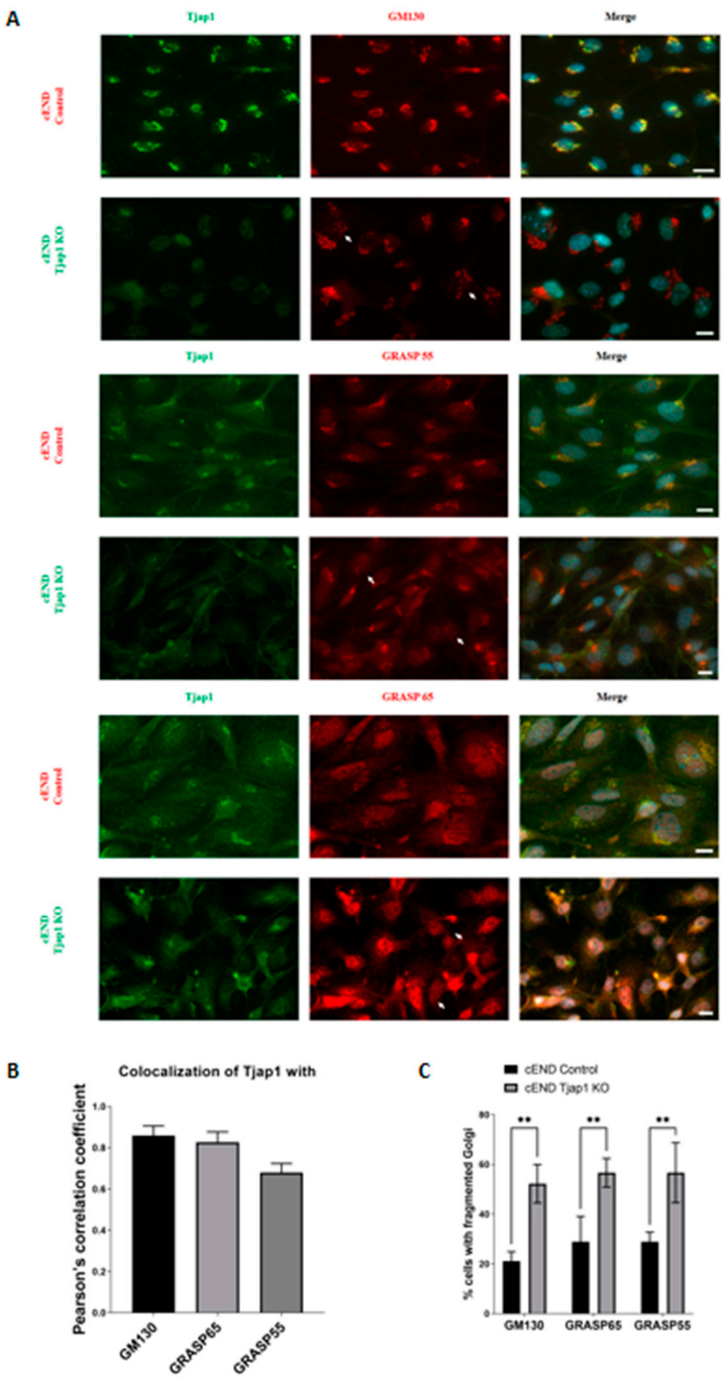

Figure S2. Tjap1 knockout induces Golgi fragmentation in cEND cells. Double immunofluorescence staining of Tjap1 (green) with GM130 (red, top panels), GRASP55 (red, middle panels), and GRASP65 (red, bottom panels) in control and Tjap1 knockout cEND cells. In control cells, GM130, GRASP55, and GRASP65 display compact perinuclear ribbon morphology. In Tjap1 knockout cells, Tjap1 staining is abolished, and all three Golgi markers exhibit dispersed punctate staining patterns (arrows), indicative of Golgi fragmentation (see also Figure. 6A–C for hCMEC/D3 cells). Nuclei were counterstained with DAPI (blue). Scale bars = 10 μm (**A**). (**B**) Quantification of the co-localization of Tjap1 with GM130, GRASP65, and GRASP55 in control cEND cells. Pearson correlation coefficients were calculated for Tjap1/GM130, Tjap1/GRASP65, and Tjap1/GRASP55. Pearson correlation coefficients were calculated using ImageJ software. Data are presented as mean ± SD, $n = 3$. (**C**) Quantification of Golgi morphology in control and Tjap1 KO cEND cells. Golgi morphology was classified for GM130, GRASP65, and GRASP55 based on blinded assessment of 30 cells per sample across three biological replicates. Statistical significance was determined by two-way ANOVA with multiple comparisons. Data are presented as mean ± SD. $^{**}p < 0.01$

Figure S3

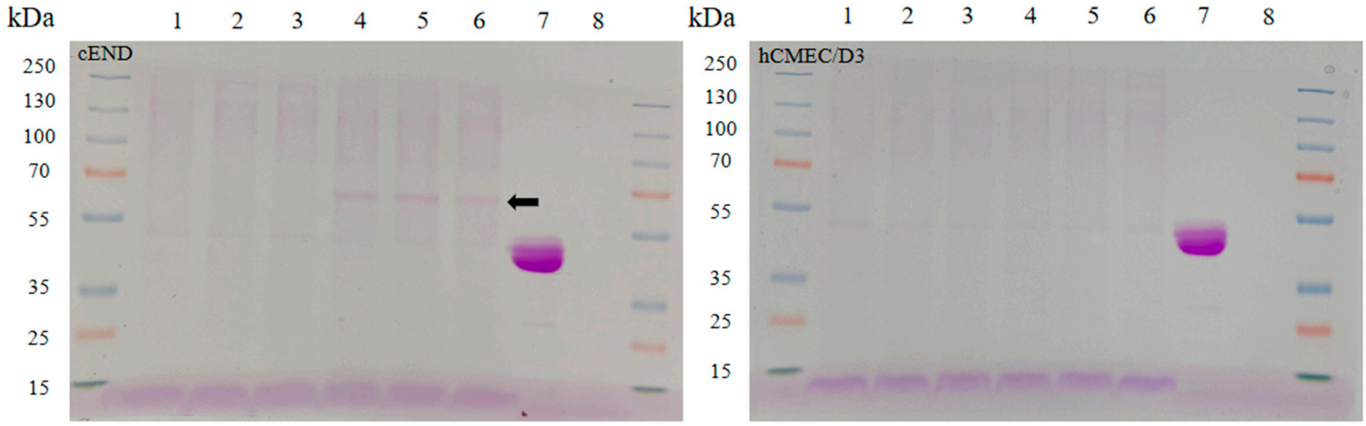

Figure S3. Glycoprotein gel staining in Tjap1 knockout BMECs. Glycoprotein gel staining of total cell lysates from control (lanes 1–3, n=3) and Tjap1 knockout (lanes 4–6, n=3) cEND cells and hCMEC/D3 cells. Lane 7 is the positive control; lane 8 is the negative control. In cEND cells, Tjap1 knockout samples show a distinct glycoprotein band at approximately 55–70 kDa (arrow), which is absent in the controls. No differences in the glycoprotein band pattern were observed between control and Tjap1 knockout hCMEC/D3 cells.

Figure S4

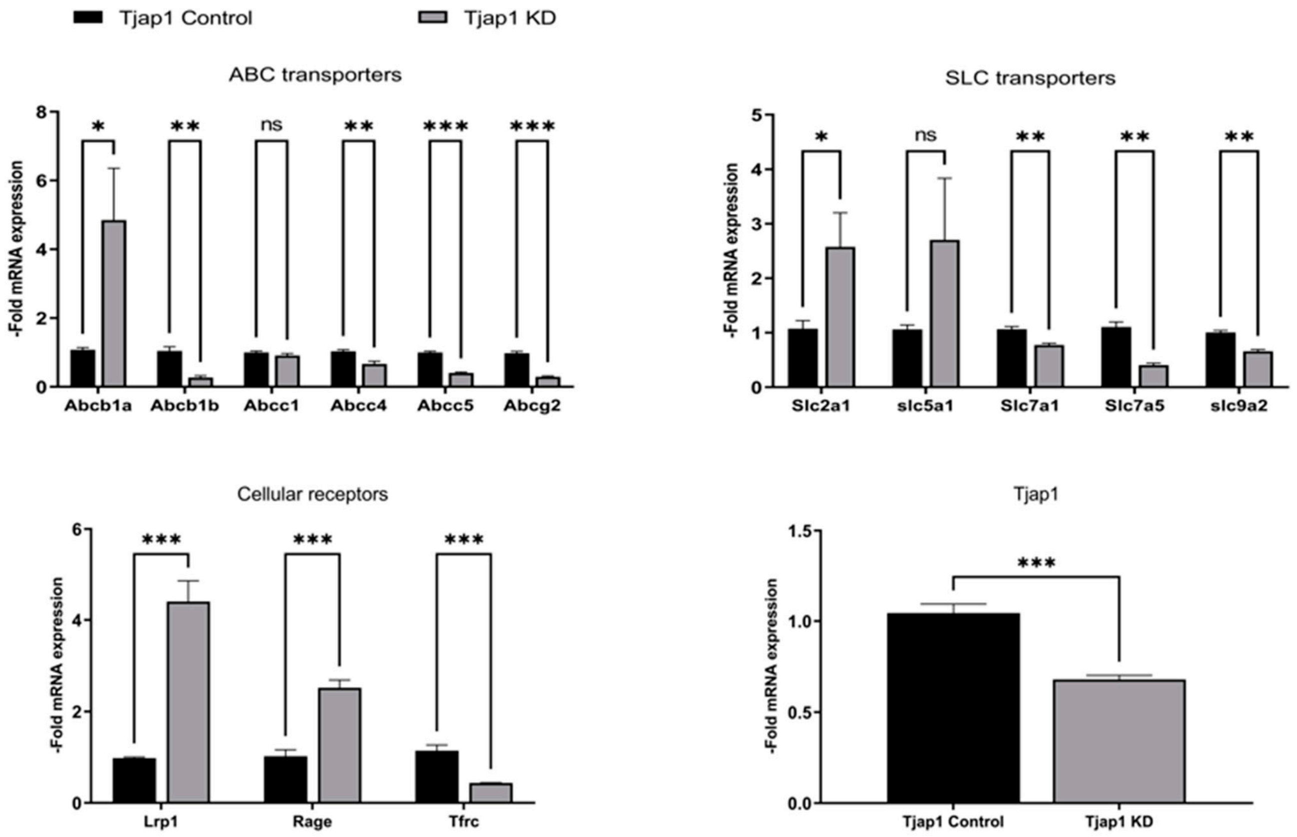

Figure S4. mRNA expression of drug transporters and cellular receptors in cEND Tjap1 knockdown cells. Quantitative real-time PCR analysis of ABC transporters, SLC transporters, cellular receptors, and Tjap1 in cEND Tjap1 knockdown (Tjap1 KD, gray bars) and control (Tjap1 Control, black bars) cells. Target gene expression was normalized to the endogenous control and presented as fold change relative to control (mean ± SD; $n = 3$). The majority of genes showed expression trends consistent with those observed in the Tjap1 knockout model (Figure. 7), with the exception of Abcb1a, Abcg2, Slc2a1, and Slc9a2, which displayed divergent responses between the knockdown and knockout conditions. *p < 0.05; **p < 0.01; ***p < 0.001; ns, not significant.
